# Reduced Variability of Auditory Alpha Activity in Chronic Tinnitus

**DOI:** 10.1155/2014/436146

**Published:** 2014-05-19

**Authors:** Winfried Schlee, Martin Schecklmann, Astrid Lehner, Peter M. Kreuzer, Veronika Vielsmeier, Timm B. Poeppl, Berthold Langguth

**Affiliations:** Department of Psychiatry and Psychotherapy, University of Regensburg, Universitaetsstrasse 84, 93053 Regensburg, Germany

## Abstract

Subjective tinnitus is characterized by the conscious perception of a phantom sound which is usually more prominent under silence. Resting state recordings without any auditory stimulation demonstrated a decrease of cortical alpha activity in temporal areas of subjects with an ongoing tinnitus perception. This is often interpreted as an indicator for enhanced excitability of the auditory cortex in tinnitus. In this study we want to further investigate this effect by analysing the moment-to-moment variability of the alpha activity in temporal areas. Magnetoencephalographic resting state recordings of 21 tinnitus subjects and 21 healthy controls were analysed with respect to the mean and the variability of spectral power in the alpha frequency band over temporal areas. A significant decrease of auditory alpha activity was detected for the low alpha frequency band (8–10 Hz) but not for the upper alpha band (10–12 Hz). Furthermore, we found a significant decrease of alpha variability for the tinnitus group. This result was significant for the lower alpha frequency range and not significant for the upper alpha frequencies. Tinnitus subjects with a longer history of tinnitus showed less variability of their auditory alpha activity which might be an indicator for reduced adaptability of the auditory cortex in chronic tinnitus.

## 1. Introduction

Subjective tinnitus is characterized by the conscious perception of a sound in the absence of a corresponding physical source. This auditory phantom sound is usually described as a pure tone, a hissing, or a roaring noise. Most of the people suffering from tinnitus report that the tinnitus sound is an ongoing and continuous perception which is typically more prominent in silent environments. Resting state measures in a silent environment should therefore be a useful tool to investigate the aberrant brain activity associated with the tinnitus. Comparison of resting brain activity of tinnitus patients and healthy controls under silent conditions should reveal the abnormal brain activity that is related to both tinnitus perception and tinnitus-associated distress.

Tinnitus-related alterations of resting state activity have indeed been demonstrated by several studies using electroencephalographic (EEG) and magnetoencephalographic (MEG) recordings. Changes in auditory areas of tinnitus sufferers comprise enhanced gamma activity [1–4], enhanced slow wave activity [1, 4–6], and reduced alpha activity [5]. The relation between these changes of spontaneous brain activity and the tinnitus has been further strengthened by longitudinal studies providing evidence that a temporary or long-lasting reduction of tinnitus symptoms is associated with a normalization of this abnormal brain activity. For instance, Adamchic and colleagues showed that reduced tinnitus severity after coordinated reset treatment relates to reduced delta and gamma power in temporal areas [7]. Kahlbrock and Weisz observed reduced auditory delta power during episodes of residual inhibition [8]. Müller and colleagues demonstrated increased auditory alpha power following successful tinnitus reduction with repetitive transcranial magnetic stimulation (rTMS) [9] and normalization of the delta and alpha power by means of neurofeedback has been shown to reduce tinnitus symptoms [10, 11].

In the current study, we sought to further investigate alpha activity in auditory areas of subjects with chronic tinnitus perception. It has been hypothesized that the oscillatory activity of the alpha frequency range (8–12 Hz) in sensory regions of the human brain might represent a gating mechanism for incoming information that is not relevant to the subject and is therefore actively suppressed [12, 13]. It has been proposed that this reduced alpha power reflects a state of desynchronized neuronal networks, which is associated with auditory attention [14, 15]. In a normal hearing participant, this desynchronized state would be temporary and would enable neuronal couplings driven by correlated stimulus attributes. In tinnitus, however, the persisting desynchronization may be a consequence of ongoing auditory attention.

A study on visual attention demonstrated an increase of alpha power in parietooccipital regions ipsilateral to the attended side together with a decrease of alpha power at the contralateral side [16]. Furthermore, Thut and colleagues showed that the amount of this prestimulus hemispheric alpha lateralization is correlated with the reaction time of the participants [17]. In a somatosensory discrimination task, Haegens et al. were able to demonstrate that prestimulus alpha lateralization is associated with the cued target location and the reliability of the cue [18]. In a cross-modal paradigm in which subjects had to switch between visual and auditory targets, Mazaheri and colleagues recently showed that the prestimulus alpha activity switches between the respective sensory brain areas depending on the stimulus modality [19]. Taken together, all these results indicate an involvement of alpha oscillations in the active suppression of irrelevant and potentially distracting sensory information.

This view on alpha activity is complemented by another line of research investigating spontaneous fluctuation of cortical alpha activity. Romei and colleagues measured the spontaneous alpha fluctuation over visual areas and applied transcranial magnetic stimulation to evoke phosphenes. Trials with a conscious perception of the phosphene were associated with reduced visual alpha activity in the visual cortex [20] which suggests an enhanced excitability of the visual cortex during moments with low alpha power. Similarly, another study showed that the detection of visual stimuli near the perceptual threshold was more reliable during episodes of alpha desynchronisation than during episodes of alpha synchronisation [21].

Our knowledge about the underlying neuronal mechanisms for these spontaneous fluctuations in the resting state is currently still relatively limited. Nevertheless, evidence is accumulating that the variability of brain activity from one moment to the other is of functional importance for the central nervous system [22]. Measuring the variability of the blood oxygen level-dependent (BOLD) signal, Garrett and colleagues demonstrated that the moment-to-moment variability increases when participants are engaged in a task rather than being in the resting state. Furthermore, participants that are performing the task faster than average usually show larger variability of their BOLD signals [23]. In another study investigating the variability of EEG phase synchronization in children with acute traumatic brain injury (TBI), Nenadovic et al. reported that children with greater variability have higher chances for recovery from TBI [24]. Measurements of brain signal variability in other pathologies like Alzheimer's disease [25] and autism [26] also show large reductions, when patients are compared with healthy control groups.

A conceptual framework for these findings is provided by the notion that variability in cortical processing is an essential feature of learning [27]. According to this theory cortical map expansion occurs during learning processes for increasing processing capacities in order to enable replication with variation. Analogous to a Darwinian mechanism the behaviourally most useful circuit is then selected and consolidated [27].

Taken together, it can be hypothesized that disease-related alterations of neuronal plasticity should be reflected by alterations in the variability of neuronal activity. Reduced variability may indicate a reduction of the dynamic range of brain response and impaired neuroplastic capacity. Here we aimed to investigate whether the moment-to-moment variability of auditory alpha activity in tinnitus is altered as compared to controls. For this purpose, we used magnetoencephalographic recordings in the resting state and compared signal variability between subjects with chronic perception of tinnitus and healthy controls. Our hypothesis was that the variability of auditory alpha activity is reduced in individuals with tinnitus.

## 2. Materials and Methods

### 2.1. Participants

Data of 42 subjects who participated in different studies [28–31] were analysed retrospectively. All participants were right-handed according to the Edinburgh Handedness Inventory [32]. Twenty-one of the participants reported an ongoing perception of tinnitus for more than 6 months (mean duration: 4 years; standard deviation: 3.3 years; range: 5–12 years). Tinnitus-related distress was measured with the German version of Tinnitus Questionnaire [33] average distress of 21.5 points (standard deviation: 16.8; range: 3–59). The mean age of the tinnitus group was 44.4 years (standard deviation: 14.8 years; range: 22–69 years; 6 female). The data of the tinnitus group was compared to an age- and gender-matched healthy control group (mean age: 43.7 years; standard deviation: 15.3 years; range: 22–69 years; 6 female). All participants gave informed written consent and the study was approved by the ethical review board of the University of Konstanz.

### 2.2. Data Acquisition

Spontaneous brain activity was recorded using a whole-head MEG system with 148 magnetometer (MAGNES TM 2500 WH, 4D Neuroimaging, San Diego, USA) at a sampling rate of 678.17 Hz or 2034.51 Hz and a hard-wired high pass filter of 0.1 Hz. For data analysis, all data were downsampled to 600 Hz. Participants were instructed to relax in supine position with eyes open and fixating a point at the ceiling of the measuring chamber and not to engage in any deliberate mental activity. Five minutes of resting state data were analysed.

### 2.3. Data Analysis

The Matlab-based FieldTrip toolbox was used for analysing the MEG data [34]. Prior to data analysis, the MEG channel positions were realigned towards standard magnetometer positions for each individual subject [35]. A discrete Fourier transform (DFT) filter was used to reduce the 50 Hz line noise. The continuous data set was cut into epochs of two seconds and those epochs containing artefacts were manually excluded from data analysis. Following the artefact correction, we selected randomly a number of 90 epochs (i.e., 180 seconds of resting state data) from the remaining trials in order to make sure that the same amount of MEG data was used for all subjects.

We calculated the time-frequency representation of the spontaneous recordings from 1 to 100 Hz with an increment of 0.5 Hz. For each of the 90 trials, the MEG data was multiplied with a Hanning window before applying the fast Fourier transform.

### 2.4. Coefficient of Variation Computation

In order to measure the moment-to-moment variability of alpha power, we calculated the coefficient of variation (CV) across the 90 trials for each individual data set and each sensor [22]. The coefficient of variation for the frequency *f* and the trial *t* is defined as the ratio of the standard deviation *σ*_*f*,*t*_ to mean *μ*_*f*,*t*_:

(1) $$\begin{array}{c} \text{C}{\text{V}}_{f,t}=\frac{\sigma_{f,t}}{\mu_{f,t}}. \end{array}$$

Thereby, the coefficient of variation expresses an unbound measure of the variability which is independent from the magnitude of the mean.

### 2.5. Statistical Analysis

Statistical analyses including the mixed models analysis of variance (ANOVA) were carried out using the open source R statistical software package available at http://www.r-project.org/ including the* nlme* library. Comparison between the tinnitus and the control group was performed with a mixed models ANOVA allowing a random intercept for each participant.

## 3. Results

### 3.1. Alpha Power Reduction in Tinnitus

**Figure 1:**
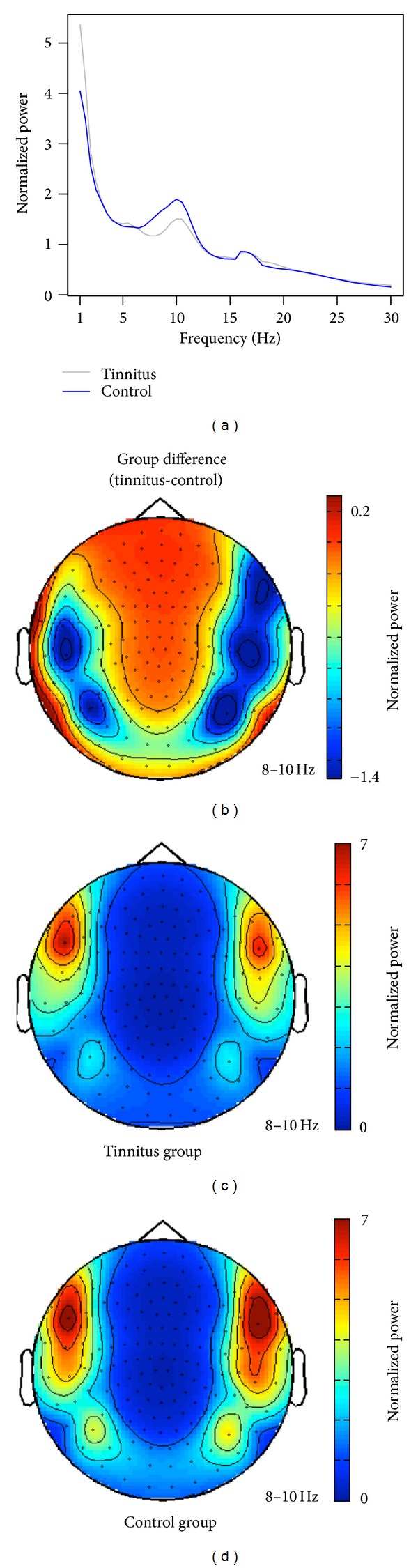
Normalized power. (a) illustrates the normalized global power averaged over all sensors. (b)–(d) show the topographical distribution of the low-frequency alpha power (8–10 Hz) for the group difference (tinnitus minus control, (b)), the tinnitus group (c), and the control group (d).

**Figure 2:**
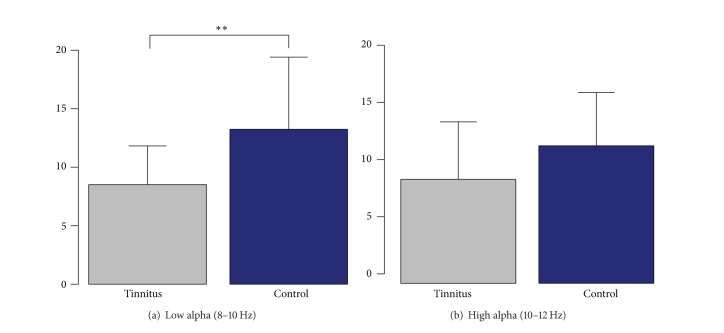
Alpha power group comparison. The bar plots demonstrate the group difference for the temporal alpha power for the lower alpha band from 8 to 10 Hz (a) and the upper alpha band from 10 to 12 Hz (b). The group difference is only significant for the lower alpha frequency range with *P* = 0.0036.

In the first step of the analysis, we intended to reproduce the finding of reduced alpha activity in chronic tinnitus as proposed by previous studies [5]. While the study by Weisz et al. only investigated the reduction in the source space, we here show that the effects are also significant on the sensor level.  Figure 1(a) shows the normalized power spectrum for the tinnitus and the control group averaged over all sensors. In Figures 1(b)–1(d) we illustrate the topographical map for each group and the group difference. For the analysis of the spectral power over auditory regions, regions of interest (ROI) were defined in order to cover the brain regions showing the strongest difference in alpha desynchronization between the tinnitus and the control group. Therefore, the ROI selection was based on the group difference in the 8–10 Hz frequency range (Figure 1(b)). Spectral power of the left and the right temporal areas were averaged for the analysis. The same ROIs were used for all of the following analyses. The analysis was done for the lower alpha band (8–10 Hz) and the upper alpha band (10–12 Hz) separately. A linear mixed models analysis of variance (ANOVA) for the lower alpha band revealed a significant group difference with *F*(1,40) = 9.58 and a *P* value of *P* = 0.0036. The ANOVA for the upper alpha band revealed with *F*(1,40) = 3.86 and *P* = 0.056 a trend towards significance. The group differences for the lower and upper alpha power are depicted in Figures 2(a) and 2(b).

### 3.2. Variability of Auditory Alpha Activity

**Figure 3:**
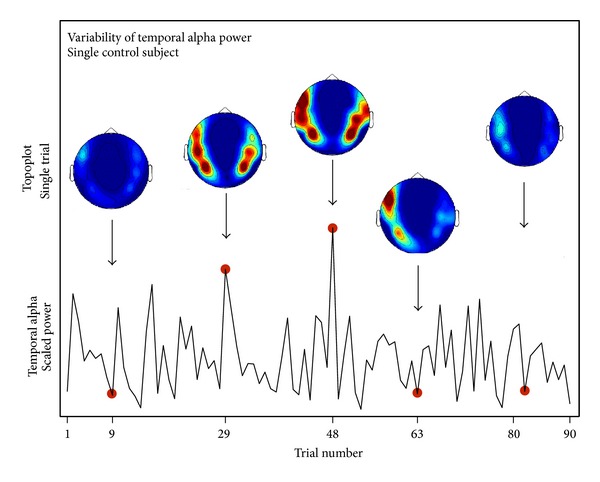
Illustration of the alpha moment-to-moment variability. Data are shown for an example control subject. Bottom: the variability of the temporal alpha activity is shown for the 90 trials. Top: topographical maps are plotted for five selected trials.

In order to analyse the variability of alpha power, we calculated a spectral analysis for each single trial. To illustrate the alpha variability we selected an example control subject and plotted the temporal alpha power for all 90 trials in Figure 3. Topoplots for selected trials are shown above. Please note that this graph does not necessarily show a continuous timeline of temporal alpha activity since some trials in between might have been rejected during artifact correction and only 90 trials have been randomly selected. It rather demonstrates the relatively large variability of alpha activity from one trial to the other.

**Figure 4:**
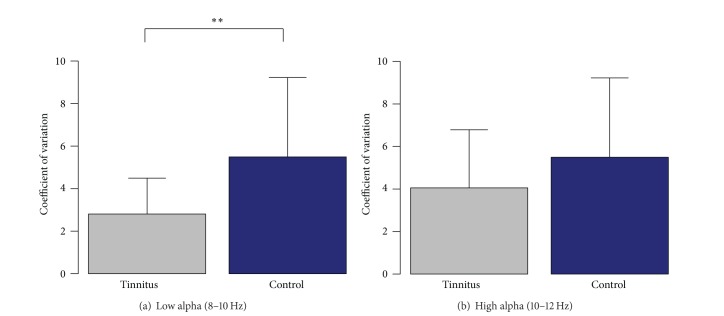
Alpha variability group comparison. The bar plots demonstrate the group difference for the variability of the temporal alpha power. Coefficients of variation are shown for both groups, for the lower and upper alpha band separately. Group differences are only significant for the lower alpha band from 8 to 10 Hz with *P* = 0.004.

In order to measure this variability, coefficients of variation (CV) have been calculated for each subject. Statistical comparison of the CV in both groups indicated a strong reduction of temporal alpha variability in the tinnitus group. The analysis was done for the lower alpha band (8–10 Hz) and the upper alpha band (10–12 Hz) separately. A linear mixed models analysis of variance (ANOVA) for the lower alpha band revealed a significant group effect with *F*(1,40) = 9.04 and a *P* value of *P* = 0.004. For the upper alpha band, the group difference was not significant with *F*(1,40) = 1.90 and *P* = 0.18. Group differences for both alpha bands are illustrated in Figure 4.

### 3.3. Longer Tinnitus Duration Is Associated with Reduced Auditory Alpha Variability

**Figure 5:**
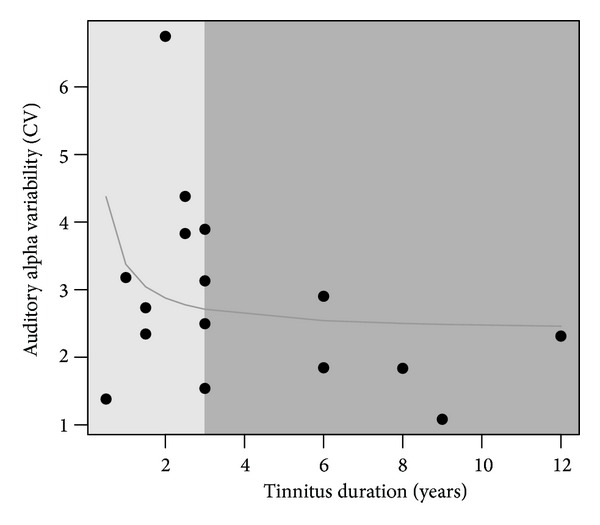
Association between tinnitus duration and alpha variability. Patients with a longer duration of tinnitus show lower levels of auditory alpha variability (8–10 Hz). A median split was calculated and revealed significant difference in the variability between patients with a long and a short duration of tinnitus with *P* = 0.038.

Further analysis of the alpha variability in the tinnitus group suggested a nonlinear relationship between the variability and the duration of the tinnitus. In subjects with a shorter duration of tinnitus we measured larger alpha variability than in subjects with longer tinnitus duration (see Figure 5). A nonlinear function was fitted to the data explaining the auditory alpha variability by 1/tinnitus duration + a, with an estimate for a of 2.38 (*t* = 6.28, *P* < 0.001). Furthermore, a median split was used to divide the group in tinnitus subjects with a shorter (less than 3 years) and longer history of tinnitus (more than 3 years). A Welch two sample *t*-test revealed a significant lower variability measures for the group with the longer tinnitus duration: *t* = 2.3, *P* value = 0.038. No association was found between the alpha variability and the tinnitus-related distress (*P* > 0.7) or the age of the subjects (*P* > 0.2).

## 4. Discussion

In the current study, we were able to repeat previous results by demonstrating reduced alpha activity in temporal regions in people with tinnitus as compared to healthy controls. In addition to former studies we differentiated in the current analysis between lower and higher alpha power and found evidence that this power reduction is more pronounced in the lower alpha frequency range (8–10 Hz) than in the upper alpha frequency range (10–12 Hz). Furthermore, we were able to show that the moment-to-moment variability of auditory alpha activity is significantly decreased in chronic tinnitus subjects. Again, this effect was more prominent in the lower than in the upper alpha frequency range. Moreover, alpha variability was more reduced in patients with a longer history of tinnitus.

Oscillatory activity in the alpha frequency range can be detected in all sensory areas and is by far the strongest oscillation that can be observed in the human brain [5, 15, 17–19]. It has been shown that episodes with enhanced alpha activity in sensory areas are characterized by reduced excitability in the respective sensory modality, while episodes with low alpha activity (i.e., alpha desynchronization) are associated with enhanced neuronal excitability of this area [20, 21, 36, 37]. In this context, electro- und magnetoencephalographic recordings of auditory activity over sensory areas can be interpreted as a measurement of a neuronal mechanism gating sensory information processing. Increases in alpha power recordings can therefore indicate suppression of sensory input that is currently not needed or even distracting, while reductions of alpha power suggest increased excitability of the sensory area for a more precise perception of potentially important sensory input. The link between enhanced neuronal excitability and reduced alpha power is currently not well established. Recently, it has been shown that the locally enhanced neuronal excitability can be also characterized by increased functional coupling with remote brain areas ([38]; see also Weisz and Obleser for a theoretical framework [39]), meaning that the respective sensory area is “ready” to receive information from distant brain regions via already established functional connections. Therefore, it might be that the alpha desynchronization is just one indicator of the enhanced neuronal excitability; the integration in a distributed brain network might be another. How this state of enhanced excitability in the auditory areas is triggered in tinnitus remains a debate. Several explanations are possible and might also depend on individual patient characteristics: (1) top-down attention to the auditory stream might trigger this state (e.g., in patients that routinely “check” if their tinnitus is still there), (2) a mismatch between the auditory phantom perception and the environment without a physical source for it might enhance the excitability in order to dissolve this mismatch, or (3) bottom-up mechanisms might also trigger regularly and/or constantly the excitability state. Here, we used the reduced temporal alpha activity as a marker for the enhanced neuronal excitability. The recordings were done during resting state in a quite environment with no relevant auditory stimulation. During this resting state, we recorded strong alpha activity over temporal areas in healthy control subjects indicating reduced excitability of the auditory cortex. In tinnitus patients, however, we recorded reduced alpha activity over auditory areas indicating enhanced neuronal excitability.

With this study we showed that auditory alpha activity is variable and fluctuates from one moment to the other. The variability was significantly reduced in the tinnitus group. This result is in line with other research showing dynamic changes of brain activity under rest [22, 40, 41]. The dynamic change of alpha activity in sensory areas reflects thereby a variability of states with enhanced and reduced excitability. It has been hypothesized that this variability is beneficial to the system insofar as that it increases the dynamic range permitting more different responses to a broader range of incoming stimuli which finally leads to greater adaptability [22]. The current results show that the variability of auditory alpha activity is reduced for the tinnitus group. Furthermore, in recordings of tinnitus subjects with longer tinnitus duration even less variability was detected. Due to the cross-sectional design of the study we cannot distinguish whether the reduced variability reflects the predisposition to develop tinnitus or the consequence of tinnitus. Thus, if the reduced moment-to-moment variability of brain activity represents a trait-marker reflecting reduced adaptability of the nervous system, one could conclude that only subjects with reduced alpha variability can enter the state of chronic tinnitus perceiving the phantom sound for many years. Tinnitus subjects with greater variability might be able to adapt to the tinnitus, which results in a spontaneous remission of the symptoms. Therefore, we do not see tinnitus subjects with great signal variability and a long history of tinnitus. If this hypothesis is true, the alpha variability should represent an indicator for spontaneous tinnitus remission.

The second explanation favours neuroplastic changes of the auditory cortex as a consequence of the chronic tinnitus perception over the years. The continuous auditory phantom perception might attract the attention to the auditory stream leading to an ongoing enhancement of excitability in the auditory cortex. This long-term potentiation might lead to plastic changes in auditory areas that finally reduce the variability of auditory alpha activity in the long run. Whether reduced alpha variability in tinnitus represents the predisposition or the consequence of tinnitus or in other words a trait or state marker should be addressed by longitudinal studies.

This pilot study which investigates for the first time the moment-to-moment variability of oscillatory brain activity in tinnitus patients has several limitations. First, the analysis focussed on alpha activity in temporal brain areas, because the reduced alpha activity in the auditory cortex is the most robust neuroimaging finding in tinnitus. However, alterations in other frequency bands [1–4] and in other brain areas [42–44] have been documented as well. Thus further studies should investigate variability in other frequency bands and other brain areas.

The variability measure that we used in this study was normalized to the mean power. Since we observed both reduced mean alpha power and reduced variability in the tinnitus group we can exclude that our finding of reduced variability is an artefact resulting from an increase in the mean alpha. Nevertheless, the used procedure is just one possibility to quantify the variability of neuronal oscillations.

In this study the control group was age- and gender-matched, but the groups were not matched for comorbidities of tinnitus like hearing loss, depression, or hyperacusis, which were not assessed in the whole study population. Therefore, we cannot rule out hearing loss, depression, or hyperacusis as alternative explanations for the reduced alpha variability. Therefore, further studies are needed which control for those potential confounding factors.

Our study has also revealed that both the reduction of alpha power and alpha variability was mainly driven by low alpha activity (8–10 Hz). This finding is new and somewhat unexpected and requires confirmation by investigations in independent samples.

## 5. Conclusions

The current study supports the idea of reduced auditory alpha activity in chronic tinnitus patients. Based on the concept that alpha activity reflects the level of inhibitory influence on sensory regions this finding can be interpreted as enhanced excitability of the auditory cortex in tinnitus. Furthermore, we showed that the auditory alpha activity in healthy controls is dynamic and varies within the range of seconds. The moment-to-moment variability of auditory alpha in tinnitus subjects is significantly reduced with a tendency that subjects with a longer tinnitus duration show less variability. This might be an indicator for reduced adaptive potential of the auditory cortex in tinnitus patients and—if confirmed by further studies—has important implications for understanding the pathophysiological underpinnings of tinnitus. Moreover, the reduced variability might represent a potential therapeutic target for neuromodulatory treatment approaches, for example, by auditory [45] or brain stimulation [46].
